# Head Trauma in Pycnodysostosis: An Imagiological Challenge

**DOI:** 10.7759/cureus.95536

**Published:** 2025-10-27

**Authors:** Catarina Faria Tavares, Inês Silva Costa, Francisco Ruas, Helena Pereira

**Affiliations:** 1 Pediatrics Department, Unidade Local de Saúde (ULS) de Viseu Dão-Lafões, Viseu, PRT

**Keywords:** bone dysplasia, cathepsin k, osteosclerosis, pediatric head trauma, pycnodysostosis, skull fracture

## Abstract

A 10-year-old girl with a known diagnosis of pycnodysostosis presented to the emergency department after a traumatic head injury. Physical examination revealed a fluctuant triangular deformity in the parieto-occipital region, raising suspicion of a skull fracture. Computed tomography (CT) demonstrated heterogeneous bone density with radiated sclerosis of the parietal bones, consistent with her underlying condition, and a partial discontinuity of the lambdoid suture without signs of an acute fracture. A conservative, watchful approach was undertaken with complete recovery. This case highlights the diagnostic challenges posed by pycnodysostosis in differentiating acute trauma from chronic skeletal abnormalities.

## Introduction

Pycnodysostosis is a rare autosomal recessive skeletal dysplasia caused by pathogenic variants in the *CTSK* gene, which encodes cathepsin K, an enzyme essential for osteoclastic bone resorption. Its deficiency leads to defective bone remodeling, resulting in dense but brittle bones. Clinically, the condition is characterized by diffuse osteosclerosis, short stature, acro-osteolysis of distal phalanges, and increased bone fragility [1,2].

Although fractures are common, cranial trauma represents a particular diagnostic challenge in these patients. The coexistence of osteosclerosis and fragility often produces imaging findings that can mimic acute fractures, making it difficult to distinguish between chronic skeletal remodeling and new traumatic lesions [3,4]. Pycnodysostosis should be differentiated from other sclerosing bone dysplasias, such as osteopetrosis, which typically causes foraminal narrowing and bone marrow failure, features absent in pycnodysostosis [5].

Recognizing this condition in the acute setting is critical, as its paradoxical combination of dense yet fragile bones can lead to misinterpretation of imaging findings and unnecessary interventions following minor trauma. Awareness of these diagnostic pitfalls in emergency care settings underscores the clinical significance of the present case.

## Case presentation

A 10-year-old girl presented to the emergency department 12 hours after sustaining a traumatic head injury to the parieto-occipital region. She complained of localized pain below the impact site. Her medical history included pycnodysostosis, under follow-up at a tertiary hospital for short stature and recurrent pathological fractures.

**Figure 1:**
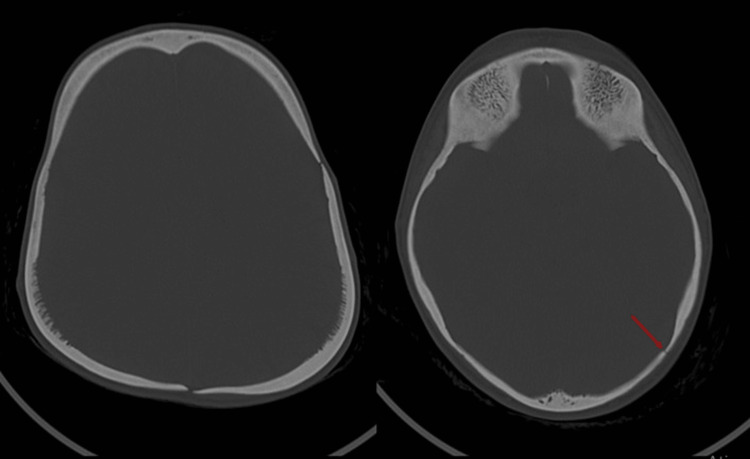
Axial CT view of the head showing heterogeneous bone density and sclerotic radiations of the parietal bones. Arrow indicates the regions of sclerotic thickening.

**Figure 2:**
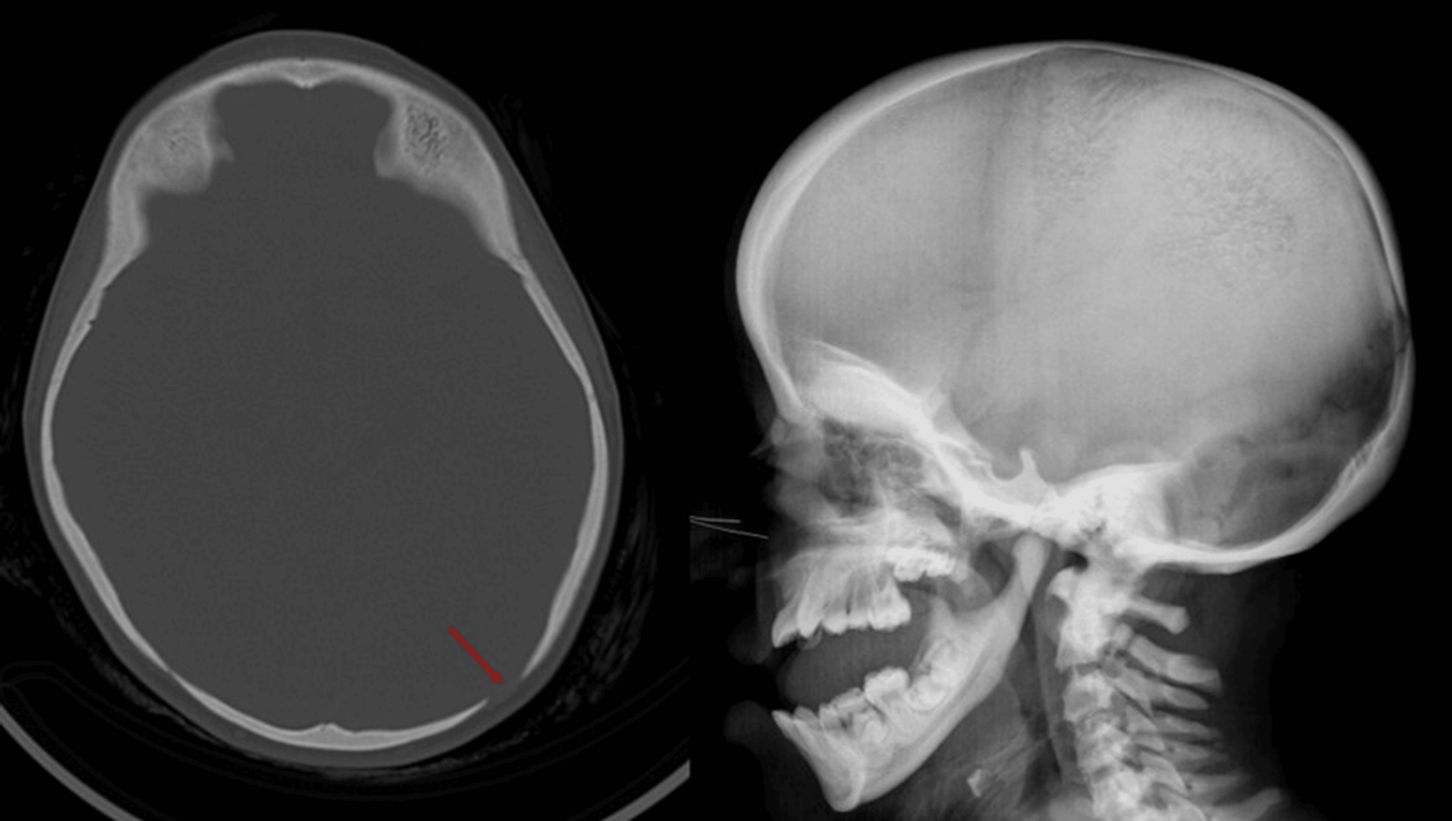
Mid-axial CT section demonstrating a widened gap between bone structures near the lambdoid lesion, with corresponding sagittal X-ray view. Arrow highlights the suture irregularity.

**Figure 3:**
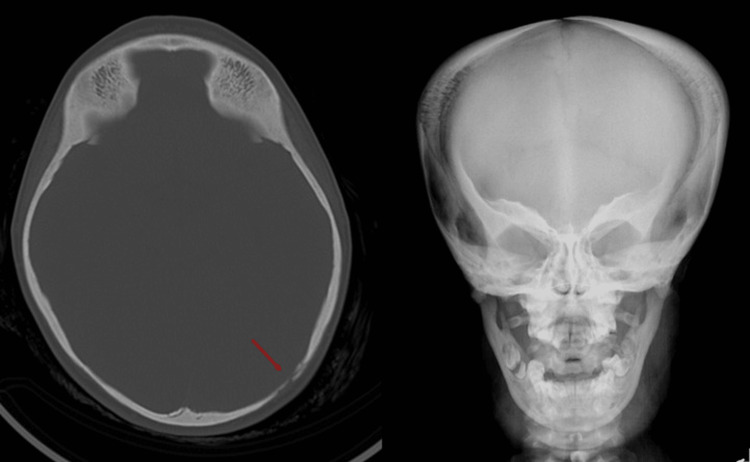
Lower axial CT slice demonstrating heterogeneous bone density and irregular sclerotic margins, with corresponding coronal radiographic view. Arrow marks the deformity margins.

Physical examination revealed a fluctuant triangular deformity at the site of impact, not previously noted in earlier evaluations. Cranial computed tomography (CT) showed heterogeneous bone density with radiated sclerosis of the parietal bones (Figures 1-3), findings consistent with the patient’s chronic bone condition. However, due to incomplete fusion of the posterior sagittal suture and a partial discontinuity along the lambdoid suture with sclerotic margins, a subtle acute fracture remained a differential radiologic consideration.

After discussion with the radiology team, these findings were interpreted as characteristic of pycnodysostosis, without evidence of a recent fracture. The patient was managed conservatively with analgesics and observation, with an uneventful course and follow-up planned at her referral hospital. Differential diagnoses such as osteopetrosis and craniodiaphyseal dysplasia were considered but excluded based on the absence of foraminal narrowing, bone marrow failure, or craniofacial overgrowth. The imaging findings, clinical history, and genetic confirmation supported the diagnosis of pycnodysostosis as the underlying cause of the observed features.

## Discussion

The imaging findings in pycnodysostosis directly reflect the underlying molecular defect. Cathepsin K deficiency impairs osteoclastic degradation of type I collagen, resulting in accumulation of unresorbed bone matrix and diffuse osteosclerosis. This abnormal remodeling produces bones that appear radiologically dense but are structurally fragile, explaining both the chronic sclerotic changes and the predisposition to fractures after minimal trauma.

In the emergency setting, misinterpreting these chronic sclerotic patterns as acute fractures may lead to unnecessary hospital admissions, imaging, or immobilization. Recognizing the molecular basis and characteristic imaging features of pycnodysostosis is therefore crucial to guide appropriate management and avoid overtreatment.

Head trauma in children is common and ranges from minor bumps to serious brain injuries, accounting for a large proportion of pediatric emergency admissions. Although most cases are mild, children are more susceptible to intracranial injury even after seemingly minor trauma. Epidemiologically, head injuries represent approximately 10%-20% of pediatric emergency visits, and only a small percentage require hospitalization [3,4]. Nonetheless, careful assessment is crucial, as subtle findings can signal significant underlying injury.

Warning signs of serious head trauma include loss of consciousness, repeated vomiting, severe or worsening headache, drowsiness, confusion, seizures, limb weakness, imbalance, or clear fluid from the ears or nose. In this case, none of these were present. However, the underlying diagnosis of pycnodysostosis introduced an additional diagnostic challenge, as the patient was predisposed to skeletal fragility and atypical imaging findings.

Pycnodysostosis is a rare skeletal dysplasia with an estimated prevalence of 0.13 per 100,000 individuals. The condition is characterized by diffuse osteosclerosis, persistent open sutures and fontanelles, multiple Wormian bones, and acro-osteolysis of the distal phalanges [1,2]. Craniofacial abnormalities include a hypoplastic mandible with an obtuse angle and absent or underdeveloped paranasal sinuses, particularly the frontal sinuses [2,5].

On cranial CT, these features are seen in greater detail, with diffuse calvarial thickening and increased bone density, open sutures, and sclerotic margins. Unlike osteopetrosis, pycnodysostosis does not cause foraminal narrowing or bone-marrow failure [6]. Three-dimensional reconstructions often reveal underdevelopment of the mandibular angle and characteristic facial contour abnormalities. These imaging findings reflect impaired bone resorption due to cathepsin K deficiency and help differentiate pycnodysostosis from other sclerosing bone dysplasias [6,7].

In the present case, the paradoxical coexistence of increased bone density and mechanical fragility, which is characteristic of pycnodysostosis, contributed to diagnostic uncertainty following minor cranial trauma. Despite the sclerotic appearance of the skull, bone brittleness predisposes these patients to fractures even after minimal impact. Recognizing this clinical paradox is crucial to distinguish chronic sclerotic remodeling from an acute fracture and to avoid misinterpretation of imaging findings.

A multidisciplinary approach involving pediatricians, radiologists, orthopedists, and geneticists is therefore essential to prevent diagnostic errors and unnecessary interventions. Integrating clinical, genetic, and radiologic data allows an accurate diagnosis, minimizes invasive procedures, and ensures appropriate long-term management for patients with pycnodysostosis [7].

## Conclusions

In this case, a 10-year-old patient with genetically confirmed pycnodysostosis presented with a fluctuant triangular cranial deformity following minor head trauma. Imaging revealed heterogeneous bone density, radiated sclerosis, and partial suture discontinuity without evidence of acute fracture. The patient remained clinically stable and was managed conservatively.

These findings illustrate the diagnostic challenge of distinguishing chronic sclerotic remodeling from acute injury in patients with sclerosing bone dysplasias. A multidisciplinary approach involving radiology, pediatrics, and genetics is essential to avoid misdiagnosis and unnecessary intervention. Awareness of the characteristic imaging patterns of pycnodysostosis can guide accurate interpretation and management in emergency settings. Moreover, documenting such cases contributes to broader clinical awareness and may inform future diagnostic protocols for rare skeletal dysplasias.

## References

[1] Sánchez Lázaro JA, Linares Álvarez L (2014). Pycnodysostosis: a rare disease with frequent fractures. (Article in Spanish). Semergen.

[2] Bocchi MB, Giuli C, Farine F, Ravaioli C, Martellini S, Farsetti P, Palmacci O (2024). Pathological fractures in patients affected by pycnodysostosis: a case series. J Clin Med.

[3] Kuppermann N, Holmes JF, Dayan PS (2009). Identification of children at very low risk of clinically-important brain injuries after head trauma: a prospective cohort study. Lancet.

[4] Osmond MH, Klassen TP, Wells GA (2010). CATCH: a clinical decision rule for the use of computed tomography in children with minor head injury. CMAJ.

[5] Rovira Martí P, Ullot Font R (2016). Orthopaedic disorders of pycnodysostosis: a report of five clinical cases. Int Orthop.

[6] Zitouna K, Jrad M, Miladi M, Barsaoui M, Drissi G, Kanoun ML (2018). Traumatic cervical spine injuries in a patient with pycnodysostosis. Eur J Orthop Surg Traumatol.

[7] Taka TM, Lung B, Stepanyan H, So D, Yang S (2022). Orthopedic treatment of pycnodysostosis: a systematic review. Cureus.

